# METTL3-mediated HOTAIRM1 promotes vasculogenic mimicry icontributionsn glioma via regulating IGFBP2 expression

**DOI:** 10.1186/s12967-023-04624-3

**Published:** 2023-11-27

**Authors:** Zhangyi Wu, Nan Wei

**Affiliations:** 1 Department of Oncology, Zhejiang Hospital, No. 12 Lingyin Road, Xihu District, Hangzhou, 310013 Zhejiang China; 2 Department of Neurosurgery, Tongde Hospital of Zhejiang Province, Hangzhou, 310012 Zhejiang China

**Keywords:** Glioma, Vasculogenic mimicry, METTL3, HOTAIRM1, IGFBP2

## Abstract

**Background:**

HOTAIRM1 is revealed to facilitate the malignant progression of glioma. Vasculogenic mimicry (VM) is critically involved in glioma progression. Nevertheless, the molecular mechanism of HOTAIRM1 in regulating glioma VM formation remains elusive. Thus, we attempted to clarify the role and mechanism of HOTAIRM1 in VM formation in glioma.

**Methods:**

qRT-PCR and western blot assays were used to evaluate the gene and protein expression levels of HOTAIRM1 in glioma patient tissue samples and cell lines. The role of HOTAIRM1 in glioma cell progression and VM formation was explored using a series of function gain-and-loss experiments. RNA-binding protein immunoprecipitation (RIP), RNA pull-down, and mechanism experiments were conducted to assess the interaction between HOTAIRM1/METTL3/IGFBP2 axis. Furthermore, rescue assays were conducted to explore the regulatory function of HOTAIRM1/METTL3/IGFBP2 in glioma cell cellular processes and VM formation.

**Results:**

We found that HOTAIRM1 presented up-regulation in glioma tissues and cells and overexpression of HOTAIRM1 facilitated glioma cell proliferation, migration, invasion, and VM formation. Furthermore, overexpression of HOTAIRM1 promoted glioma tumor growth and VM formation capacity in tumor xenograft mouse model. Moreover, HOTAIRM1 was demonstrated to interact with IGFBP2 and positively regulated IGFBP2 expression. IGFBP2 was found to promote glioma cell malignancy and VM formation. Mechanistically, METTL3 was highly expressed in glioma tissues and cells and was bound with HOTAIRM1 which stabilized HOTAIRM1 expression. Rescue assays demonstrated that METTL3 silencing counteracted the impact of HOTAIRM1 on glioma cell malignancy and VM formation capacity.

**Conclusion:**

HOTAIRM1, post-transcriptionally stabilized by METTL3, promotes VM formation in glioma via up-regulating IGFBP2 expression, which provides a new direction for glioma therapy.

**Graphical Abstract:**

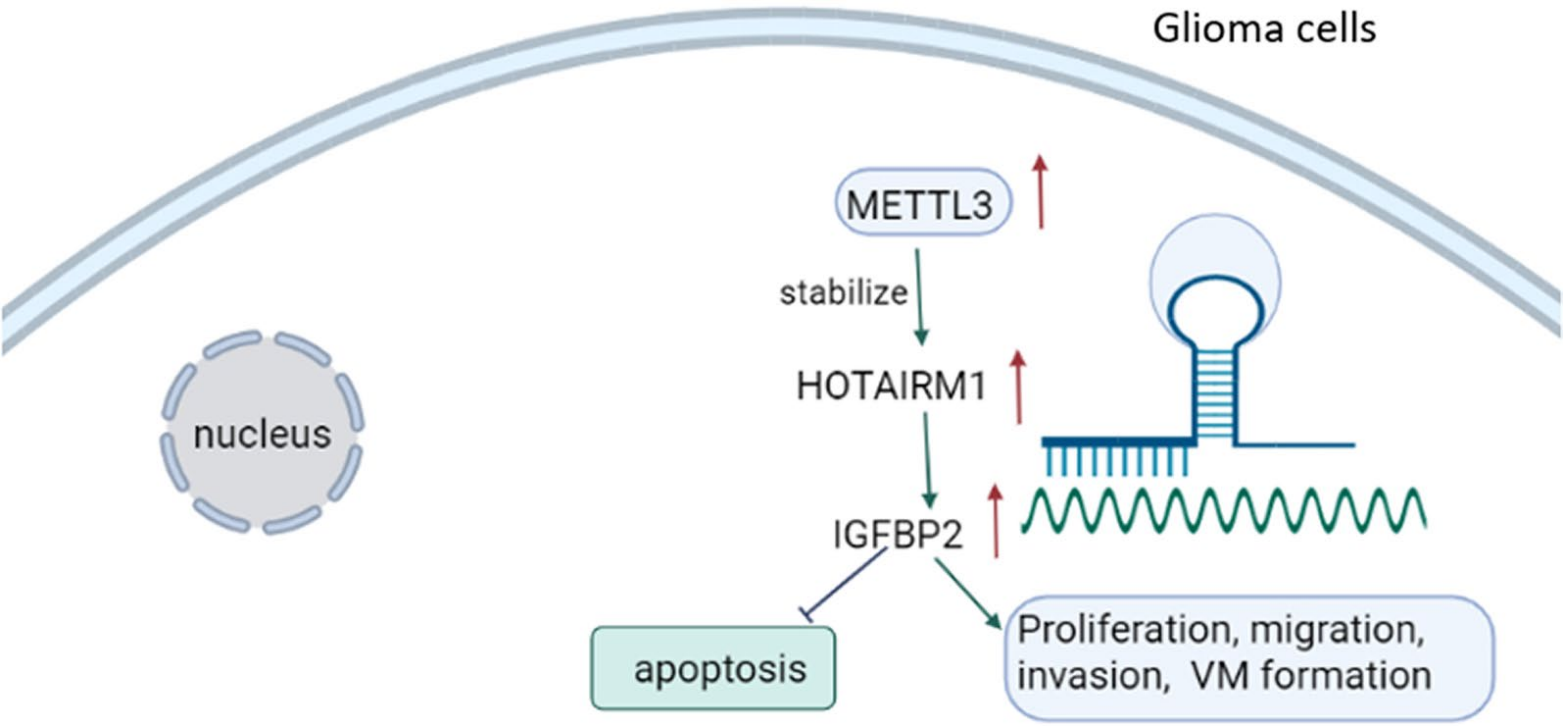

## Introduction

Glioma is the most common malignancy in the central nervous system, occupying about 40% of intracranial tumors [1]. The average lifespan of newly diagnosed patients is less than 2.5 years, and the 5 year survival is below 10%. The major glioma treatment is surgery, combined with radiotherapy, chemotherapy, immunotherapy, adjuvant therapy, and other comprehensive treatment methods [2]. Though active therapy can alleviate the symptoms of glioma patients, the overall therapeutic efficacy of gliomas remains very limited, especially for high-grade gliomas which have a high recurrence rate and a 15–26% of 2 year survival. Targeted anti-angiogenic drugs play crucial roles in glioma therapy [3]. However, anti-angiogenic therapy often produces resistance because of vasculogenic mimicry (VM), which ultimately affects the effect of anti-angiogenic therapy [4]. Thus, clarifying the mechanism related to VM formation in glioma cells is of great significance for new therapeutic insights for glioma.

VM is a novel tumor vascular model independent of angiogenesis [5]. The mechanism underlying VM formation is closely associated with the plasticity of tumor cells and tumor stem cells [6, 7]. Gliomas are highly vascularized intracranial malignancies characterized by microvascular proliferation and angiogenesis. The existence of VM is significantly related to unfavorable prognosis in glioma, and the occurrence and development of glioma can be affected by regulating vasculogenic mimicry. Liu et al. indicate that IGFBP2 promotes VM by regulating the expression of CD144 and MMP2 in gliomas [5]. Zhu et al. show that Celastrol inhibits glioma progression by blocking the PI3K/Akt/mTOR signaling pathway to regulate VM formation and angiogenesis [8]. Recently, Yi et al., have demonstrated that LOXL1-AS1-TIAR axis modulates VM formation in glioma via the miR-374b-5p/MMP14 axis [9]. Thus, exploration of the mechanism of VM is helpful to find key targets for inhibiting VM, and screening effective therapeutic drugs will provide new ideas and directions for glioma treatment.

HOX antisense intergenic RNA myeloid 1(HOTAIRM1) is a member of the HOX family, which is critically involved in glioma progression. Shi et al., indicate that HOTAIRM1 promotes glioma proliferation by regulating HOXA cluster genes high-order chromatin [10]. Previously, HOTAIRM1 has been shown to accelerate malignancy of glioma through regulating ZEB2, a competing endogenous RNA targeting molecule that interacts with miR-873-5p [11]. However, whether HOTAIRM1 is involved in glioma VM formation and its mechanism of regulating VM remain unclear. Insulin-like growth factor binding protein 2 (IGFBP2) exerts a crucial influence on glioma occurrence and development [12, 13]. Multiple studies have found that IGFBP2 plays different roles in different tumor angiogenesis [5, 13]. Recently, it was demonstrated that inhibition of IGFBP2 in U251 cells in an orthotopic mouse model significantly reduces angiogenesis mimic formation and inhibition of tumor progression, and IGFBP2 stimulates glioma cell angiogenic mimicry formation via CD144 and MMP2 up-regulation [5].

At present, the relationship between HOTAIRM1 and IGFBP2 in glioma cell and VM formation has not been investigated. Therefore, we attempted to clarify the role and molecular mechanism of HOTAIRM1 in glioma progression and VM formation capacity. Our data showed that HOTAIRM1, post-transcriptionally stabilized by METTL3, bound to IGFBP2, suggesting that HOTAIRM1 promotes VM formation in glioma via up-regulating IGFBP2 expression, providing a theoretical basis for new glioma treatment methods.

## Materials and methods

### Patient tissue specimens

Glioma patients admitted in our hospital from January 2019 to January 2021 were enrolled in this study. Glioma tissue samples were collected by surgical resections during craniotomy and confirmed by pathological examination. Patients postoperatively diagnosed as gliomas by pathologists were included in this study (n = 24). Those with the history of radiotherapy, chemotherapy, or other treatments, and/or with other tumor were excluded from the study. The pathological grades were determined according to the WHO classification [14], and the obtained glioma tissues were divided into low-grade gliomas (WHO I-II, n = 8) and high-grade glioma (WHO III-IV, n = 16). The clinical information such as age and gender of the control subjects and glioma patients are shown in Table 1, and the clinicopathological data of glioma patients are presented in Table 2. Patients with traumatic craniocerebral trauma who underwent craniotomy were used to obtain their normal brain tissue as a control (n = 12). All samples were preserved in liquid nitrogen prior to the study. All the experimental procedures were approved by the ethical committee of the Tongde Hospital of Zhejiang Province. All participants agreed and signed the informed consent prior to our study.

**Table 1:** Clinical information of the control subjects and glioma patients

Characteristics	Control subjects (%) (n = 12)	glioma patients (%) (n = 24)	P-value
Age (years)			0.35
< 50	7	10	
≥ 50	5	14	
Gender			0.24
Male	8	11	
Female	4	13	

The statistical difference was analyzed by chi-square test

**Table 2:** Correlation of HOTAIRM1 expression and clinicopathological features of glioma patients

Clinicopathological feature	HOTAIRM1 expression			
	n	Low (n = 8)	High (n = 16)	P-value
Age (years)				0.5582
< 50	10	4	6	
≥ 50	14	4	10	
Gender				0.2466
Male	11	5	6	
Female	13	3	10	
Tumor diameter				**0.0073
< 3 cm	9	6	3	
> 3 cm	15	2	13	
Tumor recurrence				*0.0321
Yes	16	3	13	
No	8	5	3	
WHO grade				**0.0094
I-II	12	7	5	
III-IV	12	1	11	

*p < 0.05, **p < 0.01 indicate statistical significance, analyzed by chi-square test

### Cell culture and transfection

Human glioma cells (U87 and U251) and normal human astrocytes NHA cells were purchased from American Tissue Culture Collection (ATCC). Cells were incubated in Dulbecco’s modified Eagle medium (DMEM, Thermo Fisher) supplemented with 10% FBS (Gibco) in a humidified incubator containing 5% CO_2_ at 37 °C. The overexpressing vectors (pcDNA3.1) of HOTAIRM1, METTL3 and IGFBP2 and PGL3-basic of IGFBP2 were constructed and synthesized by GenePharma (Shanghai, China). The vectors or the siRNA of HOTAIRM1, METTL3 or IGFBP2 were transfected into the U87 and U251cells using Lipofectamine 2000 (Invitrogen) following the manufacturer’s protocol. The siRNAs targeting HOTAIRM1, METTL3 or IGFBP2 were designed and synthesized by the Genepharma.

### Total RNA isolation and quantitative real-time polymerase chain reaction (qRT-PCR) analysis

Total RNA was isolated from glioma cells or tissue specimens by Trizol reagent (Invitrogen) according to the manufacturer’s protocol. The RNA quality and concentration were detected by Nanodrop. Then a cDNA Reverse Transcription Kit (Metabiotech) was applied for complementary DNA (cDNA) synthesis. Next, the qRT-PCR was performed to detect the expression of HOTAIRM1, METTL3 or IGFBP2 with FastStart Universal SYBR Green Master (Roche) on an Applied Biosystems 7500 real-time PCR system (Applied Biosystems). GAPDH was used as the endogenous control and the expression of HOTAIRM1, METTL3 and IGFBP2 was calculated with the 2^−△△ct^ method [15]. The primer sequences of HOTAIRM1, METTL3, IGFBP2 and GAPDH are shown below:

HOTAIRM1

F: 5′-GAAAGCGTTTGATTTATGAGCG-3′,

R: 5′-GACTATGGCTGGTTTCTGG-3′;

METTL3

F: 5′-AGACTATCTCCTGGCACTC-3′,

R: 5′-GTTTCCAAGGGTGATCCAG-3;

IGFBP2

F: 5′-GCCCTCTGGAGCACCTCTACT-3′,

R: 5′-CATCTTGCACTGTTTGAGGTTGTAC-3;

GAPDH

F: 5′-CCTCCTGTTCGACAGTCAG-3′,

R: 5′-CCATACGACTGCAAAGACC-3

### Western blot

Radioimmunoprecipitation assay (RIPA) lysis buffer (Beyotime) was used for total protein isolation from glioma tissues and cells. The total protein quality and concentration were detected using an Enhanced bicinchoninic acid (BCA) Protein Assay Kit (Beyotime). Then equal amount of protein was loaded onto SDS-PAGEs at constant voltage of 200 V, followed by transferring to PVDF membranes (Millipore). Subsequently, the membranes were blocked with 5% skim milk for 60 min at ambient temperature, and membranes were then incubated overnight at 4 °C with following primary antibodies, anti-METTL3 (ab195352, 1:1000, Abcam), anti-IGFBP2 (ab188200, 1:1000, Abcam) with GAPDH (ab9485, 1:2500, Abcam) as the loading control. Next, the membranes were incubated with the secondary antibody (ab6721, 1:2000, Abcam) for 60 min. Finally, the ECL chemiluminescent detection reagent (#32,106, 1:1, Thermo Fisher, USA) was applied to visualize the proteins, followed by analysis using ImageJ software [16].

### Cell viability

Cell Counting Kit-8 (CCK-8, Biosharp) was applied for cell viability measurement. Glioma cells with different treatment were cultured for 24 h post-transfection. Then 4 × 10^3^ cells were grown into 96-well culture plates and incubated for indicated time periods (24, 48, 72 h). Next, CCK-8 regents (10 µL) was supplemented into each well and further cultured at 37 °C for 4 h. The absorbance of the cells was detected by Infinite M200 (Tecan) at 450 nm [17].

### Cell invasion and migration assays

Cell migration and invasion abilities of glioma cells were detected using 8 μm transwell chambers (24-well insert; Costar). Then, 1 × 10^4^ cells in serum-free medium were added to the upper chamber. For lower chamber, medium containing 10% FBS were added. Cells were incubated for 48 h and cells migrated or invaded to the lower chamber membrane surface were fixated using 4% 4% paraformaldehyde (Thermo Fisher) for 15 min and dyed with 0.1% crystal violet (Sigma-Aldrich). Finally, the number of migrated or invaded cells in the lower chambers was counted under an inverted microscope (Olympus). For invasion assay, 500 ng/ml matrigel (BD, Franklin Lakes, NJ) was applied to coat the insert chamber membrane [17].

### Tube formation assay

Cold Matrigel (Corning) was diluted with the endothelial growth basal medium (EBM-2) and was used to coat the 96-well plates at 50 µl per well. After incubation at 37 °C for 60 min, cells were seeded into Matrigel-coated wells at 2 × 10^4^ cells/well [18]. Then the images were photographed after 4 h by a microscope and analyzed using ImageJ software.

### In vivo tumor xenograft mouse model

Male BALB/c nude mice (aged 4 weeks) were purchased from Charles River Labs (Beijing, China) and housed under specific pathogen-free (SPF) conditions. The animals were randomly divided into the control, Over-HOTAIRM1 and Si-HOTAIRM1 groups (n = 8 in each group). Then 1 × 10^7^ U251 cells resuspended in 0.2 ml PBS and transfected with HOTAIRM1 overexpression or silencing plasmids and parent control U251 cells were injected subcutaneously into mouse right axillary area. Tumor volume was monitored every week and evaluated with the formula: V = 0.5 × length × width^2^. After 28 days of injection, the mice were euthanized, sacrificed by cervical dislocation and tumors were removed. Tumors were then photographed, weighed, and fixated in formalin and paraffin-embedded for immunohistochemistry assays. The animal experiments were approved by the ethics committee of the Animal Experiments Ethical Inspection of Zhejiang Laboratory Animal Center.

### Terminal deoxynucleotidyltransferase (TdT)-mediated dUTP-biotin nick end labeling (TUNEL) assay

The apoptosis in the glioma tumor was examined by TUNEL assay using an in-situ cell detection kit (Roche) in accordance with the manufacturer’s instructions. Briefly, the paraffin embedded tissue slices (5 μm) were deparaffinized and rehydrated, and subsequently cultured with TUNEL reaction mixture (50 µL) for 60 min at 37 °C. DAPI was used for nucleus staining. After PBS washing thrice, the images of cells in each group were taken by a Zeiss Axioplan 2 microscope [19].

### Immunohistochemical staining

Mouse tumor tissues were fixated with 4% paraformaldehyde for 24 h, embedded in conventional paraffin, and sliced with a microtome of about 4 μm. Tissue sections were then preheated in a 65 °C incubator for 30 min and endogenous oxidase activity was blocked with a treatment of 3% hydrogen peroxide and 5% BSA. For CD34-PAS staining, the tissue sections were incubated with CD34 antibody (ab198395, 1:100, Abcam) at 4 °C overnight. The next day, the sections were placed in a 37 °C incubator for rewarming for 30 min, washed with PBS thrice for 5 min. After wiping the slices, biotin-labeled secondary antibody (ab97049, 1:1000, Abcam) was added to the tissue, incubated at 37 °C for 30 min, and rinsed with PBS for 5 min thrice. For Ki-67 and METTL3 immunohistochemical staining, the slices were incubated with anti-Ki-67 antibody (ab16667, 1:200, Abcam) and METTL3 antibody (ab195352, 1:500, Abcam) overnight, and received subsequent incubation with a secondary antibody (ab97049, 1:1000, Abcam) for 30 min at room temperature. Sections were counterstained with hematoxylin and the images were captured by a microscope. VM density was counted in five randomly selected visual fields [20].

### RNA pull down assay

RNA pulldown assay was performed with 5’-biotinylated antisense oligos. In brief, glioma cells were rinsed with cold PBS once, then cross-linked in a UV cross-linker (UVP) at 1200 mJ strength. After resuspending cells in RIPA buffer for 10 min, followed by harvesting and sonicating for 10 min, samples were centrifuged at 13,000 rpm for 20 min. Then the supernatant was added with 100 pmol probes and cultured for 2 h at 4 °C. Streptavidin Dynabeads beads (M-280, Invitrogen) were washed thrice with RIPA buffer and supplemented with 1 mg/ml BSA and 0.5 mg/ml yeast tRNA (Sigma-Aldrich, USA) and rotated for 1 h. The washed/blocked beads were then added into the supernatant with probes and then rotated for 4 h at 4 °C. After washing thrice with RIPA buffer containing 500 mM NaCl, beads were harvested with magnets (Life Technologies). The expression of IGFBP2 eluted from beads was subject to qRT-PCR analysis.

### Dual luciferase reporter assay

Potential binding sites between HOTAIRM1 and IGFBP2 were predicted by Starbase3.0 (https://starbase.sysu.edu.cn/). The IGFBP2 fragments with wild-type or mutant HOTAIRM1 binding sites were inserted into the PGL3-promoter vector. Then the siRNA of HOTAIRM1 and the PGL3-promoter vector was co-transfected into the U87 and U251 cells by Lipofectamine3000 reagent (Invitrogen) for 48 h. Dual Luciferase Reporter Assay system (Promega) was used to determine relative luciferase activity normalized to the corresponding Rellina luciferase activity following manufacturer’s instruction.

### RNA half-life detection

To analyze the RNA stability under indicated transfection, 2 mg/ml actinomycin D (Sigma-Aldrich, USA) or negative control DMSO was added into cell culture medium. Subsequently, RNA was extracted every 3 h, and HOTAIRM1 expression in glioma cells was subject to qRT-PCR analysis [21].

### Statistical analysis

GraphPad Prism 6.0 was used for statistical analyses. Each biological sample was run in triplicate and experiments were independently repeated thrice (n = 3). Data were presented as the mean ± standard deviation (SD). For data comparison between the two groups, the independent sample t-test was performed, and for multivariate comparison, the one-way analysis of variance was used followed by Tukey’s post hoc test. P-value less than 0.05 indicated the statistical significance.

## Results

### HOTAIRM1 is highly expressed in glioma cells and tissue samples

**Fig. 1:**
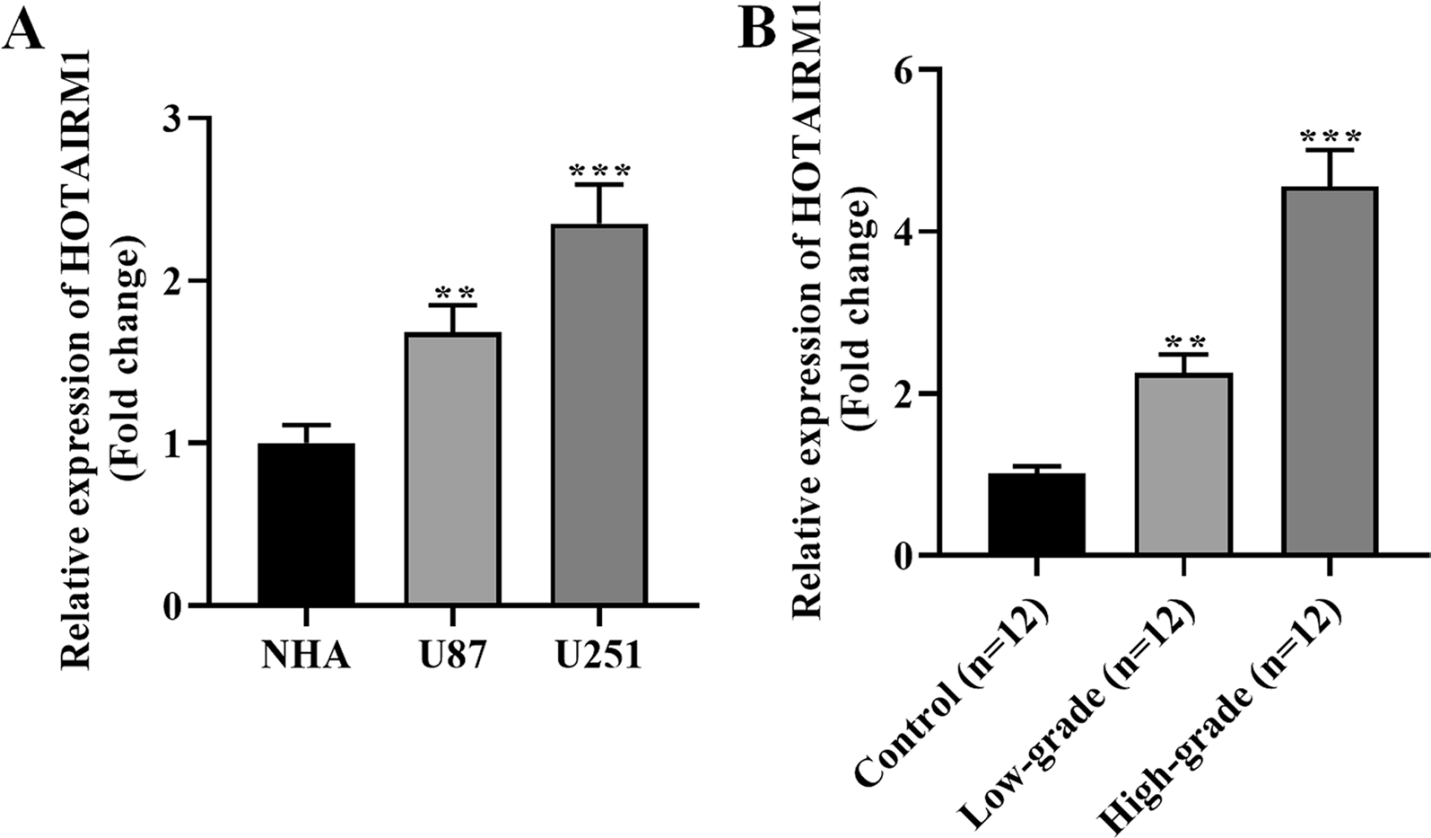
HOTAIRM1 expression is high in glioma tissues and cells. **A** qRT-PCR was used to detect HOTAIRM1 expression levels in normal human astrocyte NHA cells and glioma cell lines U87 and U251. **B** qRT-PCR examined HOTAIRM1 expression level in glioma tissue samples (n = 12 in each group). GAPDH was used as a reference control. Data are presented as the mean ± SD of three independent experiments (n = 3). **P < 0.01, ***P < 0.001

To clarify the role of HOTAIRM1 in glioma angiogenesis mimicry, we first detected HOTAIRM1 expression level in glioma cells and tissues using qRT-PCR. Relative to normal human astrocyte NHA cells, HOTAIRM1 level showed an evident increase in glioma cell lines U87 and U251 (Fig. 1A). As shown in Table 1, the control subjects and glioma patients enrolled in this study presented no statistical difference in the age and gender (P > 0.05). We also observed that HOTAIRM1 expression level was high in glioma tissue samples relative to controls, and further analysis revealed that HOTAIRM1 was more highly expressed in high-grade glioma tissue than in low-grade glioma tissue samples (Fig. 1B). Moreover, as exhibited in Table 2, the high expression of HOTAIRM1 was correlated with the tumor diameter (P = 0.0073), tumor recurrence (P = 0.0321) and WHO grade (P = 0.0094) in glioma patients. These data indicate that HOTAIRM1 may participate in glioma progression.

### Overexpression of HOTAIRM1 promotes glioma cell malignancy and VM formation

**Fig. 2:**
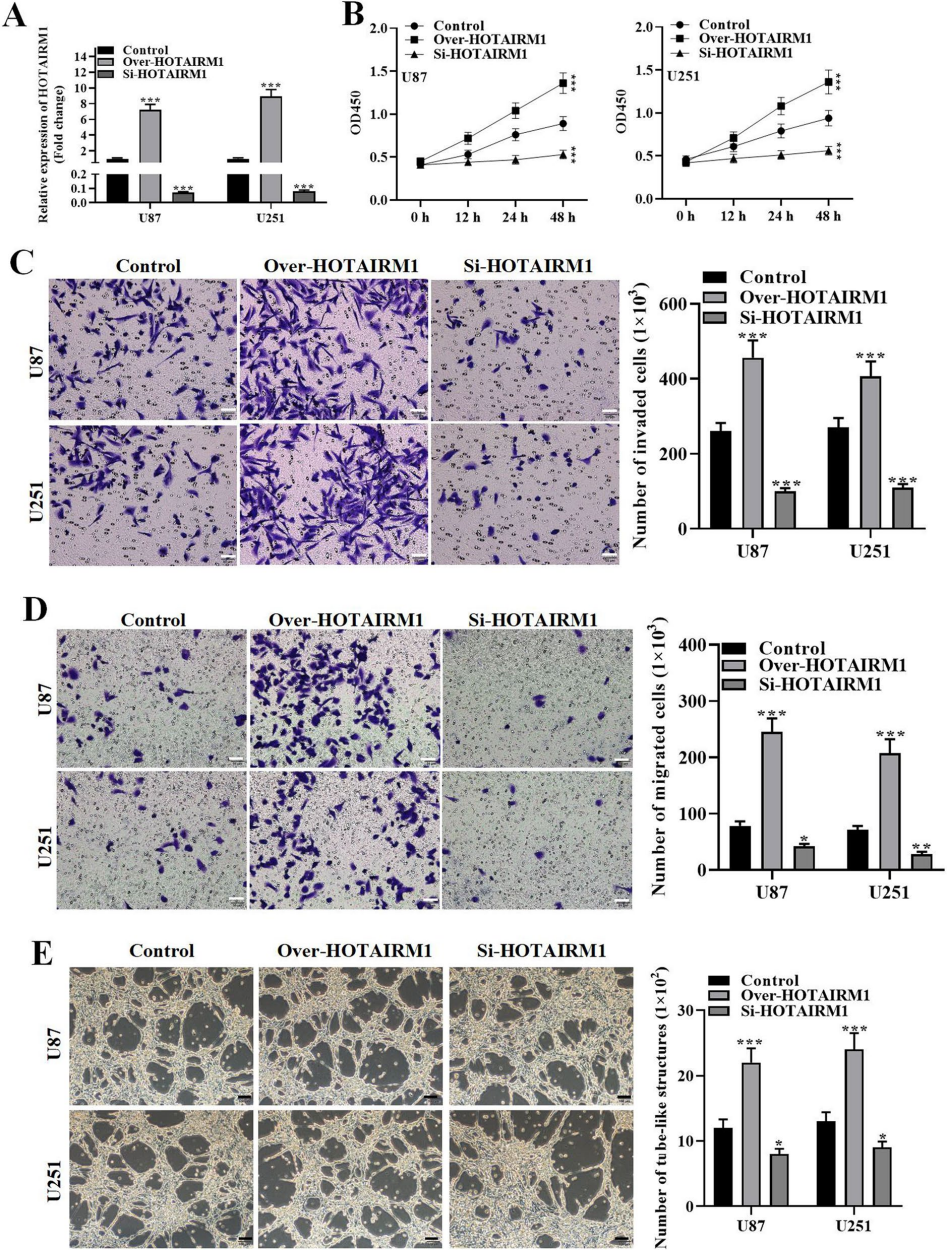
Overexpression of HOTAIRM1 promotes glioma cell proliferation, migration, invasion and VM. **A** qRT-PCR detected HOTAIRM1 expression level in glioma cells transfected with overexpressing HOTAIRM1 plasmid or siRNA. **B** CCK-8 assay was used to measure the proliferative capacity of transfected glioma cells. **C**–**D** Transwell assay detected migration and invasion capacity of transfected glioma cells (Scale bar = 50 μm). **E** Tube-formation assay evaluated VM capacity affected by HOTAIRM1 (Scale bar = 100 μm); Data are presented as the mean ± SD of three independent experiments (n = 3). *P < 0.05, **P < 0.01, ***P < 0.001

Given that HOTAIRM1 was highly expressed in glioma cells, we hypothesized that it might play a malignant role in glioma cellular processes. Thus, we conducted loss-of-function experiments. For this purpose, HOTAIRM1 was overexpressed or silenced by transfecting U87 and U251 cells with over-expressing HOTAIRM1 plasmid or si-HOTAIRM1 and empty vector control. As a result, HOTAIRM1 expression was successfully overexpressed or suppressed in transfected glioma cells (Fig. 2A). Then, CCK-8 assay was performed to assess glioma cell proliferative capability. As exhibited in Fig. 2B, cells transfected with over-HOTAIRM1 displayed increased proliferation while cells transfected with si-HOTAIRM1 showed less viability. Similarly, transwell assays demonstrated that the migratory and invaded cells were significantly increased after HOTAIRM1 overexpression and showed opposite change by silencing HOTAIRM1 (Fig. 2C and D). Moreover, tube-formation assay illustrated the elevation in VM capacity under HOTAIRM1 overexpression, which was declined under HOTAIRM1 silencing (Fig. 2E). Collectively, these results suggest that HOTAIRM1 promotes glioma cell malignant behaviors.

### HOTAIRM1 promotes glioma malignant progression and VM capacity in vivo

**Fig. 3:**
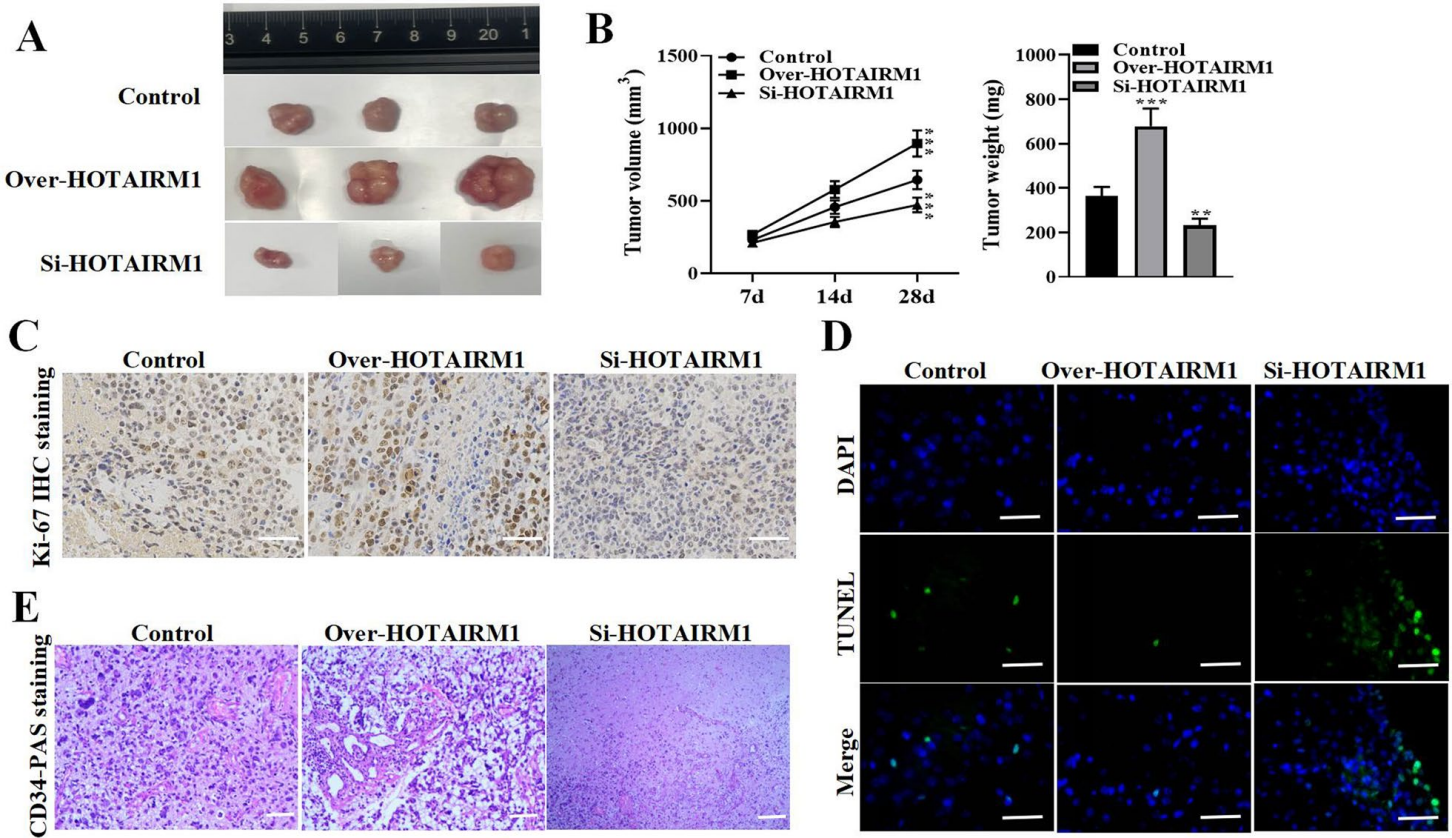
Overexpression of HOTAIRM1 promotes glioma tumor growth and VM formation in the xenograft mouse model. **A** Representative images of tumors collected from mice injected with U251 cells transfected with over- HOTAIRM1, si-HOTAIRM1or control. **B** Tumor volume was measured every 7th day, and tumor weight was detected on day 28 after injection, respectively. **C** Expression level of Ki67 in xenograft tissues from different treatment groups (Scale bar = 200 μm). **D** TUNEL assay was performed to detect cell apoptosis in different treatment groups (magnification, × 200). **E** CD34-PAS immunohistochemistry was used to assess glioma cell VM density affected in indicated treatment groups (Scale bar = 100 μm). Data are presented as the mean ± SD of three independent experiments (n = 3). **P < 0.01, ***P < 0.001

To further verify the functions of HOTAIRM1 in glioma progression in vivo, xenograft tumor experiments were conducted. The U251 cells transfected with over-HOTAIRM1, si-HOTAIRM1, or empty vector control were inoculated into nude mice, and we found that the tumor growth was facilitated in nude mice following injection with over-HOTAIRM1 than that in the control group. Notably, silencing HOTAIRM1 significantly inhibited tumor growth (Fig. 3A). Subsequently, tumor volume and weight had a significant increase under HOTAIRM1 overexpression while showed decrease under HOTAIRM1 depletion (Fig. 3B). Additionally, Ki-67 immunohistochemical staining assay revealed increased Ki-67 expression when HOTAIRM1 was overexpressed which was declined when HOTAIRM1 was silenced (Fig. 3C). TUNEL assay indicated that cell apoptosis was markedly inhibited under HOTAIRM1 overexpression whereas significantly promoted under HOTAIRM1 knockdown (Fig. 3D). Moreover, CD34-PAS immunohistochemical staining showed that glioma cell VM density was significantly increased under HOTAIRM1 overexpression and prominently reduced under HOTAIRM1 depletion (Fig. 3E). Taken together, these data demonstrate that elevated levels of HOTAIRM1 facilitates glioma tumor growth and VM formation capacity in vivo.

### HOTAIRM1 interacts and binds with IGFBP2

**Fig. 4:**
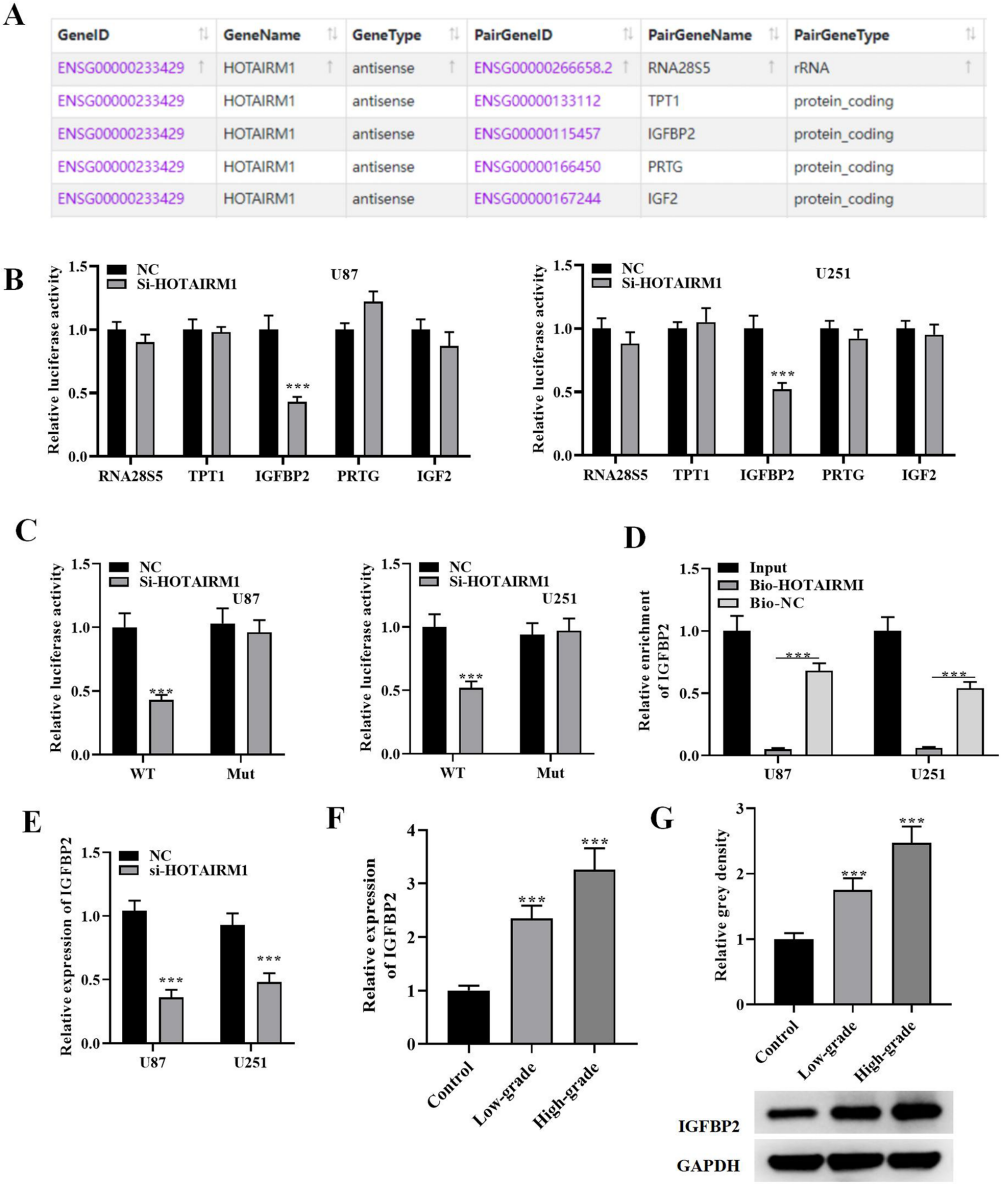
HOTAIRM1 interacts with IGFBP2. **A** The top five RNAs with binding sites to HOTAIRM1 were screened using the Starbase3.0 online tool. **B** Luciferase assay was used to detect the interaction of five candidate RNAs with HOTAIRM1. **C** Luciferase assay was used to detect the interaction of IGFBP2 and HOTAIRM1. **D** RNA pull-down assay confirmed the interaction relationship between IGFBP2 and HOTAIRM1. **E** qRT-PCR detected the expression of IGFBP2 in glioma cells after silencing HOTAIRM1. **F** qRT-PCR and **G** western blotting techniques were utilized to measure the IGFBP2 gene and protein expression levels in glioma tissues. Data are presented as the mean ± SD of three independent experiments (n = 3). ***P < 0.001

In the next step, we explored the downstream regulators of HOTAIRM1. To this end, we predicted the downstream factors that bind to HOTAIRM1 utilizing Starbase3.0 online tool. As exhibited in Fig. 4A, the top five RNAs with binding sites to HOTAIRM1 were selected for further analysis. According to luciferase activity assay results, only the IGFBP2 activity exhibited a significant reduction after knockdown of HOTAIRM1, while the luciferase activities of other four genes showed no evident alteration (Fig. 4B). Thus, IGFBP2 was chosen for further assays. Furthermore, we also found that the luciferase activities showed no significant change with mutant binding site of IGFBP2 in glioma cells after HOTAIRM1 down-regulation (Fig. 4C). Subsequently, RIP assay illustrated that both IGFBP2 and HOTAIRM1 were markedly enriched in immunoprecipitation through bio-HOTAIRM1 beads rather than control (Fig. 4D), supporting the binding relation of IGFBP2 with HOTAIRM1 in glioma cells. Moreover, we also found that HOTAIRM1 silencing induced significant reduction in IGFBP2 expression in glioma cells (Fig. 4E). We then analyzed the expression of IGFBP2 in glioma tissues. Results revealed that in comparison with healthy controls, IGFBP2 presented up-regulation in glioma tissues, and had higher level in high-grade glioma tissues than that in low-grade glioma tissues (Fig. 4F and G). These data indicate that HOTAIRM1 interacts with IGFBP2 and regulates its expression.

### HOTAIRM1 facilitates glioma cell malignancy and VM formation via regulating IGFBP2

**Fig. 5:**
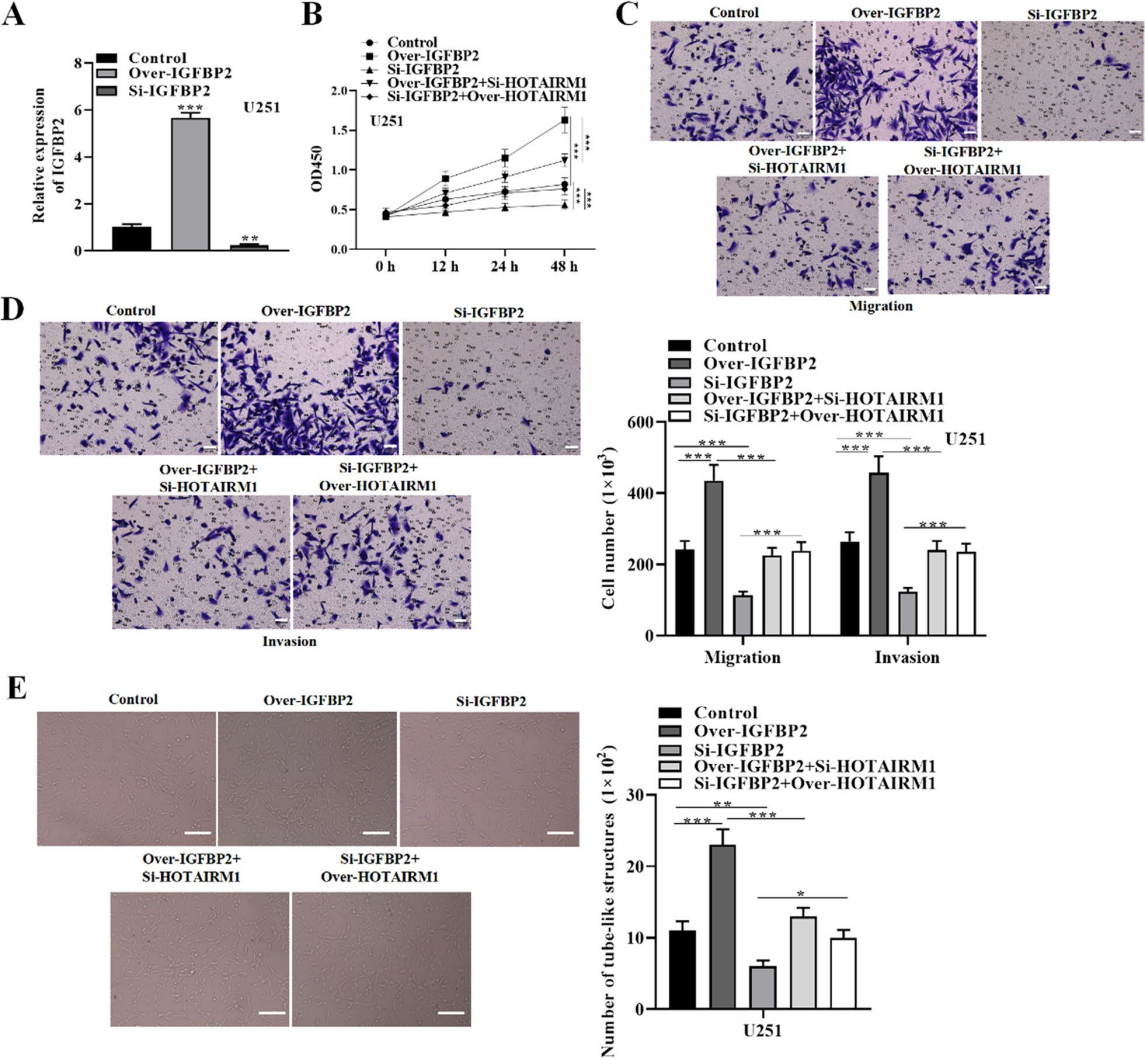
HOTAIRM1 facilitates glioma cell malignancy and promotes VM formation via regulating IGFBP2. **A** qRT-PCR was used to detect IGFBP2 expression level in glioma cells transfected with overexpressing IGFBP2 plasmid, si-IGFBP2 or control. **B** CCK-8 assay evaluated glioma cell proliferation in indicated transfected groups. **C**–**D** Transwell assay detected glioma cell migration and invasion capacity in indicated transfected groups (Scale bar = 50 μm). **E** Tube-formation assay was used to assess VM capacity in indicated transfected groups (Scale bar = 100 μm). Data are presented as the mean ± SD of three independent experiments (n = 3). *P < 0.05, **P < 0.01, ***P < 0.001

To clarify HOTAIRM1-IGFBP2 regulatory pattern in glioma cellular processes, we conducted rescue experiments. IGFBP2 was overexpressed or silenced by transfecting U87 and U251 cells with over-expressing IGFBP2 plasmid or si-IGFBP2 and empty vector control together with HOTAIRM1 overexpression or silencing plasmids. As shown in Fig. 5A, IGFBP2 was successfully elevated or repressed in glioma cells. CCK-8 illustrated that overexpressing IGFBP2 promoted the proliferation while suppressing IGFBP2 inhibited proliferation of U251 cells compared with control group (Fig. 5B). Accordingly, transwell assay indicated that overexpressing IGFBP2 facilitated U251 cell migration and invasion while showed opposite results when IGFBP2 was silenced compared with control group (Fig. 5C and D). Tube-formation showed that IGFBP2 overexpression elevated VM formation capacity while IGFBP2 silencing inhibited VM formation (Fig. 5E). Notably, the function of IGFBP2 in glioma cell malignancy and VM was found to be partially countervailed when overexpressing HOTAIRM1 in IGFBP2 silenced U251 cells or silencing HOTAIRM1 in IGFBP2 overexpressing U251 cells (Fig. 5B–E). These results highlight that overexpression of HOTAIRM1 promotes glioma cell malignancy and VM formation by up-regulating IGFBP2 expression.

### METTL3 is up regulated in glioma cells and tissue samples and stabilizes HOTAIRM1

**Fig. 6:**
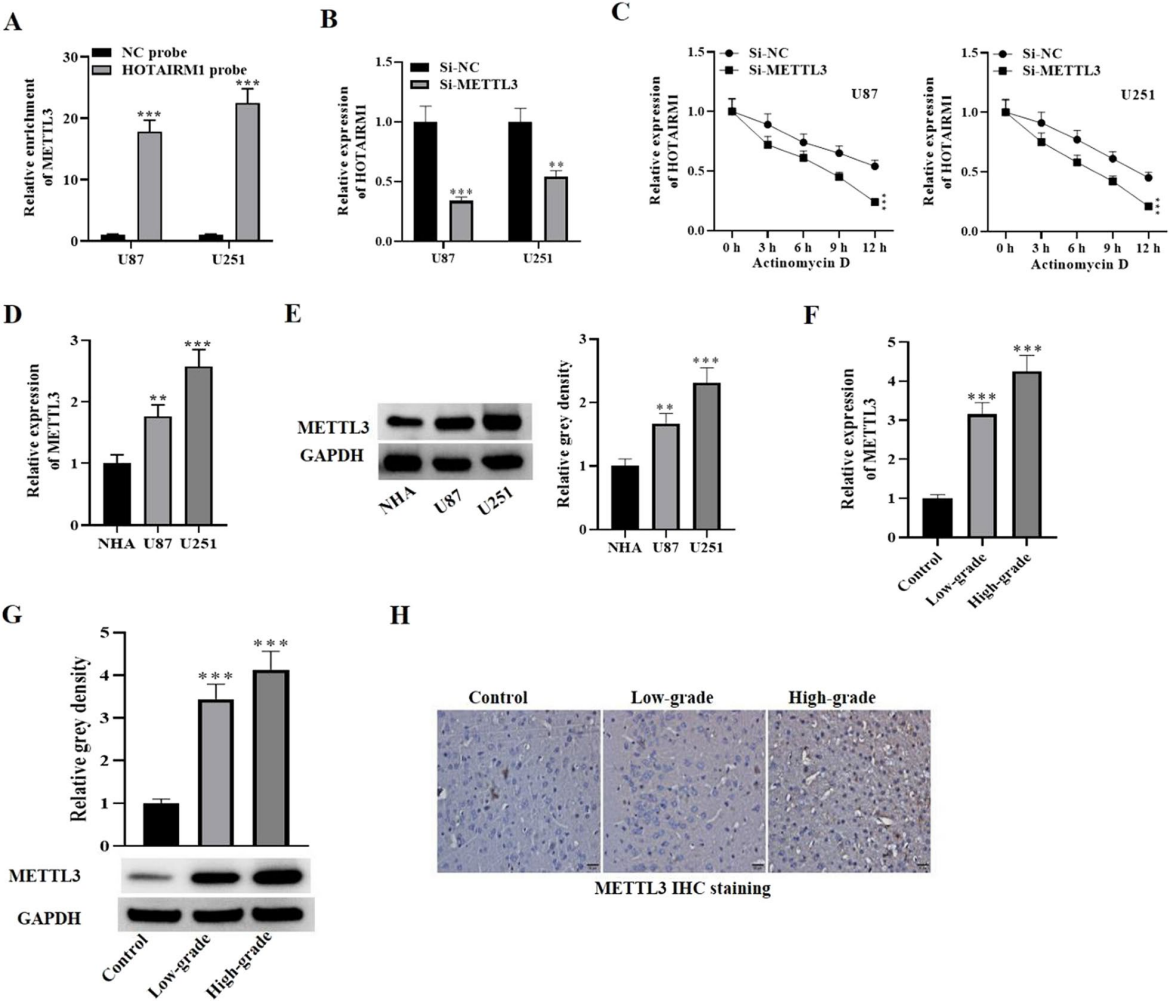
METTL3 expression is high in glioma tissues and cells. **A** RNA pull-down assay was performed using the HOTAIRM1 probe to enrich METTL3. **B** qRT-PCR was used to examine HOTAIRM1 expression levels in U87 and U251 cells transfected with either si-NC or si-METTL3. **C** qRT-PCR was performed to measure the transcriptional stability of HOTAIRM1 under METTL3 silencing in U87 and U251 cells. GAPDH was used as an internal control. **D** qRT-PCR and **E** western blot techniques were utilized to measure METTL3 expression level in glioma cells. **F** qRT-PCR and **G** western blot techniques were utilized to evaluate METTL3 expression level in glioma tissues. **H** The expression level of METTL3 in the glioma tissue sample was measured using immunohistochemistry (Scale bar = 50 μm). Data are presented as the mean ± SD of three independent experiments (n = 3). **P < 0.01, ***P < 0.001

Growing evidence in recent years has demonstrated that METTL3, an m6A methyltransferase that functions as both an oncogene and a tumor suppressor gene, plays an important role in various cancers development. Therefore, we hypothesized that HOTAIRM1 might promote glioma cell malignancy and facilitate VM formation through this mechanism. We first performed RNA pull-down assay with HOTAIRM1 probe to investigate the interaction between METTL3 and HOTAIRM1. METTL3 presented significant enrichment in the complex pulled down by HOTAIRM1 probe (Fig. 6A), suggesting that METTL3 interacts with HOTAIRM1. We then carried out rescue and stability experiments to further confirm the interaction between METTL3 and HOTAIRM1 and to investigate whether HOTAIRM1 was stabilized by METTL3. We found that HOTAIRM1 level decreased significantly when METTL3 was silenced relative to control group (Fig. 6B). Furthermore, the transcriptional stability of HOTAIRM1 remarkably decreased upon METTL3 silencing relative to NC group under treatment with actinomycin D (Fig. 6C), highlighting that HOTAIRM1 was stabilized by METTL3. Subsequently, qRT-PCR, western blotting and immunohistochemical staining were performed to measure the expression changes of METTL3 in glioma cells and tissues, respectively. As shown in Fig. 6D, METTL3 level in U87 and U251 cells presented remarkable increase in comparison to the control cells. Western blot analysis also showed a significant up-regulation of METTL3 in glioma tissues (Fig. 6E). Further analysis found that METTL3 presented up-regulation in low and high-grade glioma tissue compared to the control and its expression was higher in high-grade glioma tissue relative to that in low-grade (Fig. 6F–G). In addition, immunohistochemical staining also presented similar results in glioma tissues (Fig. 6H), further confirming elevated levels of METTL3 in glioma cells and tissues. These data suggest that HOTAIRM1 is stabilized by METTL3, and METTL3 might exert a vital role in glioma progression.

### HOTAIRMI silencing partially reverses METTL3-dependent glioma cell malignancy and VM capacity

**Fig. 7:**
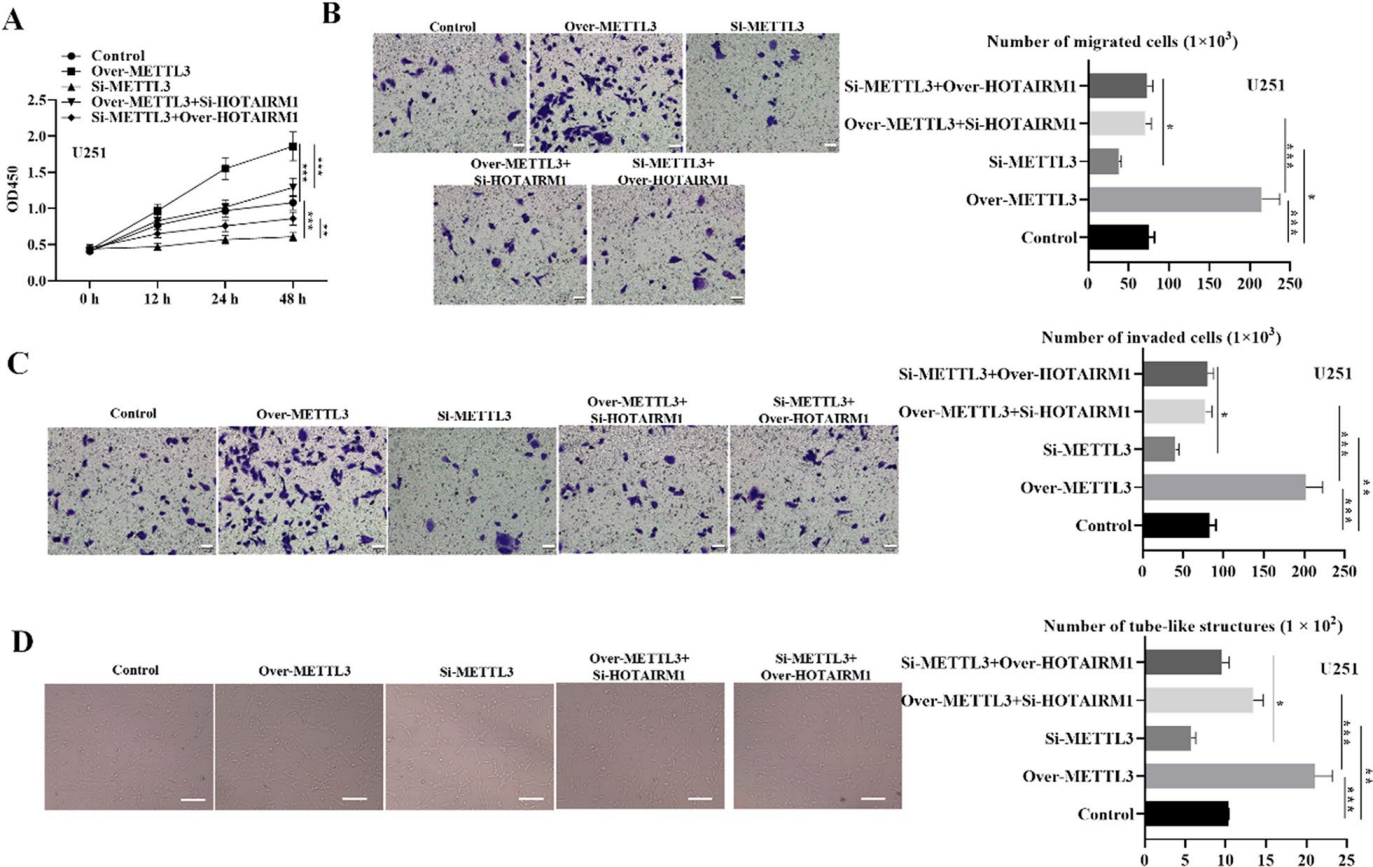
HOTAIRMI silencing partially reverses METTL3-dependent U251 cell malignancy. **A** CCK-8 assay was used to detect glioma cell proliferation in indicated transfected groups. **B**–**C** Transwell assay was conducted to evaluate glioma cell migration and invasion capabilities in indicated transfected groups (Scale bar = 50 μm). **D** VM formation capacity in indicated transfected groups was assessed by Tube-formation assay (Scale bar = 100 μm). Data are presented as the mean ± SD of three independent experiments (n = 3). *P < 0.05, **P < 0.01, ***P < 0.001

**Fig. 8:**
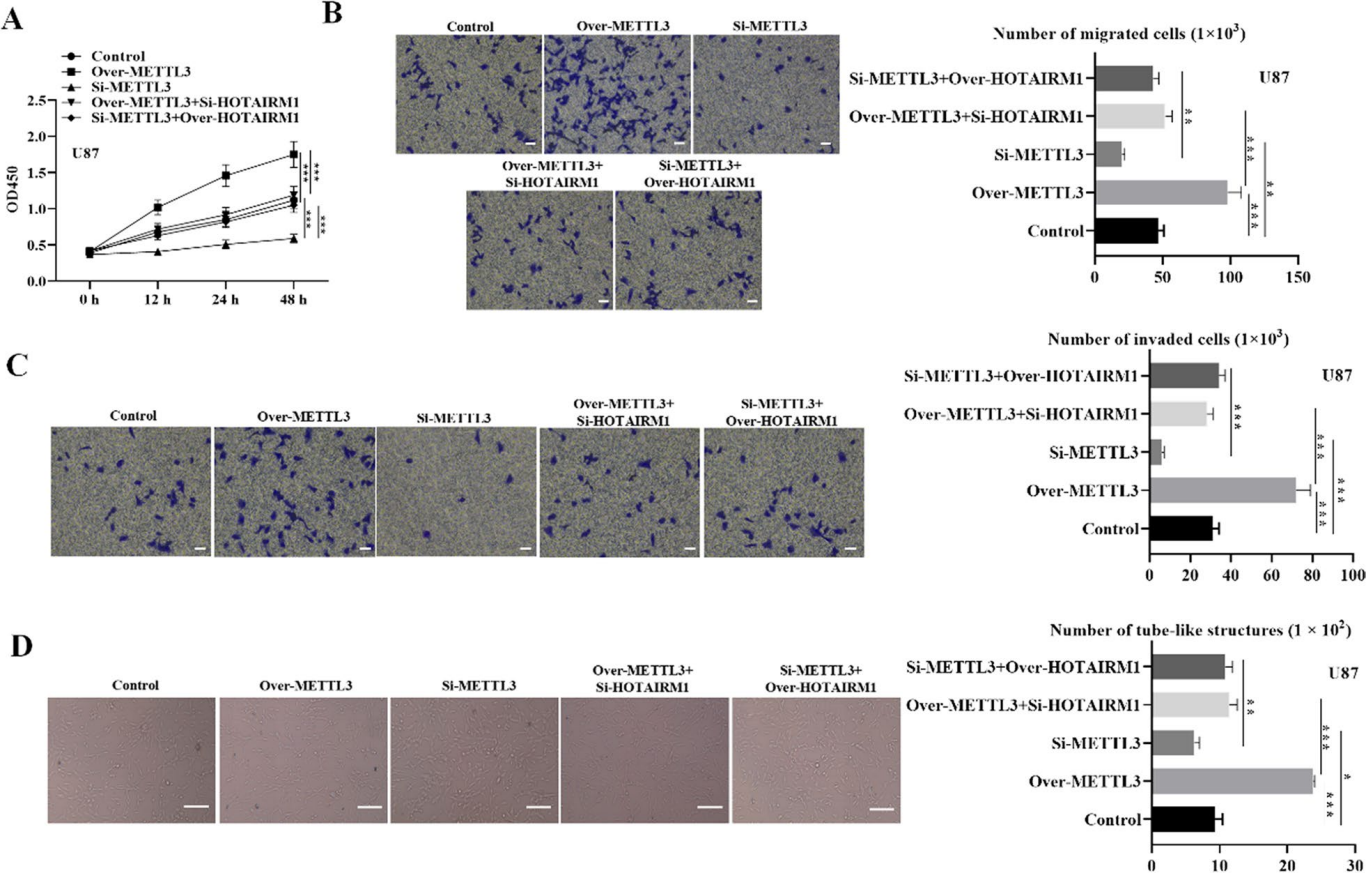
HOTAIRMI silencing partially reverses METTL3-dependent U87 cell malignancy. **A** CCK-8 assay was used to detect glioma cell proliferation in indicated transfected groups. **B**–**C** Transwell assay was conducted to evaluate glioma cell migration and invasion capabilities in indicated transfected groups (Scale bar = 50 μm). **D** VM capacity in indicated transfected groups was assessed by Tube-formation assay (Scale bar = 100 μm). Data are presented as the mean ± SD of three independent experiments (n = 3). *P < 0.05, **P < 0.01, ***P < 0.001

We then investigated the effects of METTL3 on glioma cell malignancy and evaluated METTL3-HOTAIRM1 regulatory pattern in glioma cellular processes by conducting loss-of-function experiments. Results revealed that glioma cell proliferation was significantly promoted when METTL3 was overexpressed, and glioma cell proliferation capacity was reduced when METTL3 was silenced relative to the control (Fig. 7A). Similarly, the migratory and invaded glioma cell numbers significantly increased under METTL3 overexpression while showed reduced numbers under METTL3 silencing (Figs. 7B and C and 8B and C). Furthermore, the VM capacity was markedly elevated in over-METTL3 group while inhibited in si-METTL3 group relative to controls (Figs. 7D and 8D). Additionally, we used rescue assays to explore whether METTL3 was involved in HOTAIRM1-mediated glioma cell malignancy and VM formation. Notably, HOTAIRM1 silencing reversed the impact of METTL3 overexpression on glioma cell malignancy and VM formation (Figs. 7 and 8). Collectively, these results suggest that HOTAIRM1 silencing partially counteracts METTL3 mediated glioma cell malignancy and VM formation capacity.

## Discussion

VM is significantly related to unfavorable prognosis of glioma, and the occurrence and development of glioma can be affected by regulating VM [22]. LncRNAs play crucial roles in the regulation of VM formation in diverse malignancies. However, the regulatory mechanism of HOTAIRM1 in glioma VM remains unclear [23]. In this study, HOTAIRM1 presents up-regulation in glioma cells and tissue samples and overexpression of HOTAIRM1 promotes glioma cell malignancy and VM formation capacity both in vitro and in vivo. Mechanistically, HOTAIRM1 promotes VM formation mediated by METTL3 in glioma progression via regulating IGFBP2 expression.

VM is a new tumor microcirculation model independent of endothelial cells. It has been reported that VM exists in various malignant tumors, such as liver cancer, prostate cancer, and ovarian cancer [24–26]. Gliomas are highly vascularized intracranial malignancies characterized by microvascular proliferation and angiogenesis. Multiple studies have demonstrated that VM play important roles in the glioma progression [22]. Yi et al., indicate that LOXL1-AS1 modulates VM in glioma via regulating the miR-374b-5p/MMP14 axis [9]. Zhang et al., show that exosomal miR-29a-3p from human mesenchymal stem cells inhibits migration and VM in glioma [27]. HOX gene plays an important regulatory role in the process of cell differentiation. HOTAIRM1, a member of the HOX family, is a transcriptionally active lncRNA located between the HOXA1 and HOXA2 gene sequences. Previous studies have shown that the three exons of lncR-HOTAIRM1 play important roles in gene expression and cell proliferation, as well as in glioma. HOTAIRM1 promotes malignant progression of transformed fibroblasts in glioma stem-like cells remodeled microenvironment via regulating miR-133b-3p/TGFβ axis [28]. Ahmadov et al., indicate that HOTAIRM1 facilitates tumor aggressiveness and radiotherapy resistance in glioblastoma [29]. Previously, HOTAIRM1 is also revealed to accelerate the malignant progression of glioma by regulating ZEB2, a competing endogenous RNA targeting molecule that interacts with miR-873-5p. However, the function of HOTAIRM1 in VM remains unclear. In the present study, we evaluated the role of HOTAIRM1 in VM formation and identified high HOTAIRM1 expression in glioma cells and tissue samples. HOTAIRM1 overexpression promoted glioma cell proliferation, migration, invasion and VM formation in vitro and promoted glioma tumor growth and VM formation capacity in vivo. These results indicates that HOTAIRM1 promotes glioma progression via regulating VM, suggesting it as a potential target for the design of anti-angiogenic drugs against glioma.

IGFBP2 exerts a vital impact on glioma progression [12]. Yuan et al. used tissue microarray and RNA in situ hybridization techniques and found that IGFBP2 mRNA overexpression was observed in 23.9% of the tested tumors, while in normal or edematous tissues IGFBP2 mRNA expression was not detected. Kaplan-Meier survival analysis revealed that glioma patients with high IGFBP2 expression were associated with shorter survival time than those with low IGFBP2 tumors [30]. Peng et al. demonstrated that miR-592 showed a significant down-regulation in glioma cell lines and tissue samples, and further studies revealed that overexpression of miR-592 significantly reduced cell proliferation, migration and invasiveness and arrest of cells in G0 phase and induced G1/migration in vitro by inhibiting IGFBP2 expression [30]. In fact, IGFBP2 plays a role in VM formation in glioma [5, 31]. Recently, Liu et al. have found that inhibition of IGFBP2 in U251 cells in an orthotopic mouse model could reduce VM formation and inhibit tumor progression. Mechanistic studies show that IGFBP2 stimulates glioma cell VM formation via CD144 and MMP2 up-regulation [5]. In line with these studies, we also found that IGFBP2 expression was high in glioma cells and tissues. IGFBP2 was identified to interact with HOTAIRM1. We also noticed that IGFBP2 was downregulated in glioma cells after HOTAIRM1 knockdown and IGFBP2 facilitated HOTAIRM1-triggered glioma cell malignancy and VM formation.

m6A methylation is implicated in cancer progression through regulating proto-oncogene and tumor suppressor gene expression at the level of epigenetic modification through methyltransferases and demethyltransferases [32, 33]. Several studies have demonstrated that m6A methylation participates in regulating different biological processes and affects the occurrence and development of glioma [34], Dixit et al., indicate that YTHDF2 acts as oncogene in glioblastoma Stem Cells [35]. Chang et al., reveal that METTL3 promotes the IDH-wildtype glioma malignant progression by enhancing the stability of MALAT1 that results in the activation of NF-kB signaling pathway [36]. Qiao et al. found that METTL3 in liver cancer tissues is positively correlated with VM formation. Another study showed that m6A modification of YAP1 affects the translation of YAP1 mRNA and promotes VM formation in liver cancer [37]. In our study, we uncovered that METTL3 expression was high in glioma cells and tissues. METTL3 was found to post-transcriptionally stabilized HOTAIRM1 and positively regulated its expression in glioma cells. Further analysis indicated that METTL3 depletion partially rescued the impact of HOTAIRM1 on glioma cell malignancy and VM formation. These results are in accordance with the above reported studies. The findings of our study demonstrate the oncogenic role of HOTAIRM1 in glioma progression and deepens the understanding of the underlying mechanism of HOTAIRM1 involved in glioma.

## Conclusion

In summary, our data demonstrate that HOTAIRM1 facilitates METTL3-mediated VM formation in glioma via up-regulating IGFBP2 expression (graphical abstract). Our research will provide theoretical basis to develop new treatment methods for glioma.

## Data Availability

The original contributions presented in the study are included in the article. Further inquiries can be directed to the corresponding authors.
